# Health democracy in Europe: Cancer patient organization participation in health policy

**DOI:** 10.1111/hex.12638

**Published:** 2017-11-01

**Authors:** Kyriakos Souliotis, Lily E. Peppou, Eirini Agapidaki, Chara Tzavara, Dominique Debiais, Stanimir Hasurdjiev, Francois Sarkozy

**Affiliations:** ^1^ Faculty of Social and Political Sciences University of Peloponnese Corinth Greece; ^2^ University Mental Health Research Institute Athens Greece; ^3^ Department of Hygiene, Epidemiology and Medical Statistics Centre for Health Services Research Medical School National & Kapodistrian University of Athens Athens Greece; ^4^ Europa Donna, Forum France Paris France; ^5^ Bulgarian National Patients' Organization Sofia Bulgaria; ^6^ FSNB Health & Care Paris France

**Keywords:** health policy decision making, patient‐centred care, patient empowerment, patient involvement, patient rights

## Abstract

**Background:**

Patient organization participation in health policy decision making is an understudied area of inquiry. A handful of qualitative studies have suggested that the growing number of patient organizations in Europe and their increasing involvement in policy issues do not result in high political effectiveness. However, existing research is largely country‐specific.

**Objective:**

To examine the degree and impact of cancer patient organization (CPO) participation in health policy decision making in EU‐28 and to identify their correlates.

**Methods:**

A total of 1266 members of CPOs participated in this study, recruited from a diversity of sources. CPO participation in health policy was assessed with the Health Democracy Index, a previously developed instrument measuring the degree and impact of patient organization participation in various realms of health policy. Additional questions collected information about participants' and the CPO's characteristics. Data were gleaned in the form of an online self‐reported instrument.

**Results:**

The highest degree of CPO participation was observed with respect to hospital boards, reforms in health policy and ethics committees for clinical trials. On the contrary, the lowest was discerned with regard to panels in other important health‐related organizations and in the Ministry of Health. The reverse pattern of results was observed concerning the Impact subscale. As regards the correlates of CPO participation, legislation bore the strongest association with the Degree subscale, while organizational factors emerged as the most important variables with regard to the Impact subscale.

**Conclusions:**

Research findings indicate that a high degree of CPO participation does not necessarily ensure a high impact. Efforts to promote high and effective CPO participation should be geared towards the establishment of a health‐care law based on patient rights as well as to the formation of coalitions among CPOs and the provision of training to its members.

## 1 INTRODUCTION

In contemporary health care, patients are envisioned to assume a more active role than in the past, a revolutionary paradigm known as “patient‐centred care”.^1^, ^2^, ^3^ In particular, the term describes “a partnership among practitioners and patients to ensure that decisions respect patients' desires, needs, and preferences and that patients have the education and support they need to make decisions and participate in their own care” (p. 7).^4^ Nonetheless, patient participation is not constrained on the individual level, as patients may collectively participate in decision making in various subjects, including guideline development, government policy and research agenda setting among others.^5^, ^6^, ^7^, ^8^

A number of theoretical arguments have been put forward to bolster patient participation at the collective level.^9^ Above all, it advances democratic legitimacy. Patients are affected by the consequences of certain decisions, and therefore, they should have a say in the process. In this rationale, patient participation has been shown to have an empowering effect on patients and to increase their self‐efficacy.^10^, ^11^, ^12^ Equally important is patients' experiential knowledge of a disease and its treatment, which may enhance the quality of health‐care decisions.^13^ Patients may offer solutions consonant with their preferences, contributing thereby to the prevention of mistakes and the containment of costs. Thus, the overall effectiveness and efficiency of the health‐care system are upgraded. Indeed, there are some studies to corroborate this reasoning, with evidence indicating that patient participation may promote optimal quality of care and patient safety,^14^, ^15^ curb health‐care costs 16 and enhance population health outcomes.^17^

In the context of patient‐centred care, patient participation is interwoven with patient empowerment. For example, the Patient Empowerment and Centredness Committee considers patient empowerment to be a prerequisite for patient involvement, which in turn fosters the establishment of a patient‐centred health‐care system.^18^ Indeed, published models of patient empowerment have underscored the need for patient involvement and participation.^19^, ^20^, ^21^ Empowerment also acquires a central position in the European Charter of Fundamental Rights,^22^ where citizens' right to health care is exalted. In a similar vein, many countries have launched raising awareness campaigns and have even introduced pertinent legislation. An illustration of this point is the French Act of 2 March 2002, which called for a “health democracy,” paving the way for patients to exert influence on their own health. Concomitantly, the European Commission has consistently conjured patients' rights in collaboration with various stakeholders, while a number of health and patient associations have promoted patient empowerment via various Bills of Rights or Declarations.^23^ In countries outside Europe, serious obstacles in issuing and implementing similar legislations have been documented^24^, ^25^; however, not many reports have elaborated on these issues in low‐ and middle‐income countries. At the same time, in spite of the importance of the topic, patient empowerment and participation on the meso‐level (ie regional) and macro‐level (ie national or international level) are largely understudied worldwide.^26^, ^27^

While patients' collective action has been identified as an indispensable vehicle for influencing health policy and service provision, there is a dearth of research on their associations.^12^, ^27^, ^28^, ^29^, ^30^, ^31^, ^32^ Perhaps the most well‐known study in the field is the De Montfort study in UK,^30^ which set out to explore a cross‐section of health consumer groups, their relationship with each other and their impact on national policy. It is noteworthy that the study addressed 5 disease conditions: arthritis, cancer, health and circulatory disease, maternity and childbirth and mental health. Findings corroborated that contact between health consumer groups and policymakers has risen in frequency the past years, while professional bodies and pharmaceutical companies have also included patient groups and consumers in discussions on policy proposals. Nonetheless, as the authors note, “this says little about the powers of health consumer groups either individually or collectively. It may simply be that inclusion in the policy process leads to incorporation” (p. 753).^33^ In other words, the inclusion of health consumer groups in health policy decision making may solely serve the purpose of adding legitimacy to governments, while advancing their own interests. Similarly, research from the Netherlands suggests that while the Dutch model aims to render patient organizations an equal party in health policy processes, this is not met in practice.^12^ Similarly, evidence from the Mixed Advisory Committees in Italy underscores the limited impact of users' voice on decision making by health authorities.^34^ Moreover, in a 2‐year comparative study aiming to assess the political economy and effectiveness of 500 patient associations in UK and America, Wood 27 has argued that the proliferation of patient associations in both countries has not been tantamount to high political effectiveness.

In the same study, the authors delineated the different forms of political activity and advocacy work patient organizations engage into. Newsletters and leaflets are often used to raise members' (patients' and relatives') awareness about a medical condition, new treatment techniques, available medication, existing health facilities and exemplars of good practice. Concomitantly, raising awareness activities may also target health professionals. To this end, some associations organize highly prestigious national and international conferences, contribute to the design and delivery of educational programmes and publish bulletins aimed at physicians. Lobbying of local health authorities and lobbying at the national and international level for various purposes, such as drug approval, research funding, are also common routes of political activity among patient organizations. Finally, campaigning initiatives may range from working with the government in campaigns targeting the public to exerting overt pressures in order to change government policy. In the realm of cancer, it is the advocacy work on the part of the National Coalition for Cancer Survivorship that succeeded in introducing the concept of “survivorship” as a distinct stage on the cancer control continuum in the jargon of oncology.^35^ Nonetheless, evidence on the political effectiveness of these activities on the part of patient organizations is limited.

Existing research on the impact of patient organizations on health policy decision making is largely country‐specific. The paucity of international studies is worrisome, especially in the light of the growing involvement of European institutions in health policy. Furthermore, international comparisons in health policy may facilitate the identification of “what works, why and under what circumstances, and then aggressively develop means to translate these comparative findings into new models for organizing and delivering health‐care”.^36^ In response to this literature gap, a workshop with 22 academic researchers and two representatives of patient organizations was held in Vienna in 2006.^37^ The workshop recorded high engagement of health consumer groups with policymakers and political institutions, however with marked diversity among countries. It was concluded that further comparative research is needed in Europe and patient organization activities should be analysed at the pan‐European level.

In this context, this study builds upon previous work on the development and validation of a Health Democracy Index,^38^, ^39^, ^40^ an original scale measuring the degree and impact of patient organization participation in health policy decision making. While there have been some measures assessing patient empowerment^41^ and patient participation in the micro‐level,^42^ there are no psychometrically robust tools measuring patient participation in health policy decision making from the patient perspective. On these grounds, the Health Democracy Index was developed and validated. By utilizing this tool, this study set out the following research aims:

1. To describe the degree and impact of CPO participation in health policy in EU‐28
2. To identify the organizational and contextual correlates of this participation, both in terms of its degree and impact, after controlling for individual characteristics

The study explored the aforementioned aims with regard to cancer patient organizations. The reason for addressing only one disease group rather than adopting a more disease‐general approach is justified by evidence indicating that health conditions display marked diversity in terms of their impact on the population, their priority status within a government policy, their media and public profile as well as the degree to which they are ingrained in the medical model.^30^ Furthermore, a recent study has bolstered the moderator effect of type of disease in the association between patient empowerment and therapy compliance.^43^ Cancer was chosen as the disease of interest, primarily because it constitutes a major public health concern incurring significant burden to European societies.^44^ It is among the main causes of morbidity and mortality worldwide, while its treatment cost is high.^45^ Furthermore, inequalities in accessing cancer treatment have been reported, while the need to tackle treatment barriers has been repeatedly stressed.^46^ Concomitantly, cancer has a high political profile, as it attracts great media attention and influences more people.^37^

## 2 METHOD

### 2.1 Sample

The present sample consisted of members of cancer patient organizations (CPOs). A CPO was defined as any patient group with a legal entity (formal organizations) that addressed cancer solely. To be included into the sampling list, a CPO had to meet certain criteria: (i) be active on a national level and (ii) have an accessible website. In the absence of a European list entailing existing CPOs, a sampling frame/list had to be constructed by members of the research team. To this end, patient organizations were identified through various sources: Internet search, online databases of European cancer patient organizations, registries of the Ministries of Health, direct contacts with researchers, European umbrella organizations, etc.

CPOs were identified and contacted via email or telephone. The number of individual members corresponding to these CPOs could not have been computed. To be eligible for participation, an individual should have been a member of the organization and older than 18 years old. Overall, 1.266 members of CPOs responded to the questionnaire.

### 2.2 Instrument

The self‐reported questionnaire consisted of the following sections:

#### 2.2.1 Health Democracy Index 38, 39, 40

This is an original scale measuring patient participation in health policy decision making. Earliest versions of the instrument^38^, ^39^ encompassed 8 items; however, its extended form^40^ consisted of 17 questions: 8 items tapping the degree of patient organization participation (“Degree” subscale) and 9 tapping the impact of this participation on health policy (“Impact” subscale). The 8 items corresponding to the Degree subscale enquired about patient organization participation in the following realms of decision making: reforms, panels/workshops at the Ministry of Health, panels/workshops in other important health‐related organizations, hospital boards, ethics committees in clinical trials, health technology assessment procedures (2 items, one on the economic evaluation of new treatments and methods and one on their scientific evaluation) and the national parliament. Responses on these items were made on a 7‐point scale: (i) it is not a legal requirement and it never happens, (ii) it is not a legal requirement and it rarely happens, (iii) it is not a legal requirement, but it often happens, (iv) it is a legal requirement and it never happens, (v) it is a legal requirement and it often happens, (vi) it is a legal requirement and it happens very often, and (vii) it is a legal requirement and it always happens. On the other hand, the Impact subscale enquired about the impact of this participation in the 8 aforementioned realms (8 items) as well as about the frequency by which a substantial change is observed in the content of a health policy decision as a corollary of this participation (1 item). Ratings are made on a 6‐point scale ranging from absent to very high for the former 8 items and from never to very often for the 9th question. Higher composite scores on the subscales denote higher degree and impact of participation. Both subscales displayed good internal consistency (Cronbach α = 0879 and Cronbach α = 0874, respectively). Converging evidence has substantiated the psychometric properties of the Health Democracy Index.^38^, ^39^, ^40^

#### 2.2.2 Individual characteristics

One section of the instrument entailed questions about respondents' individual characteristics. This included their socio‐demographic profile (gender, age, family status and educational attainment) and their involvement in the CPO. With regard to the latter, individuals had to rate their familiarity with the disease and their knowledge about treatment/the country's health‐care system/country's reimbursement processes (very low‐low‐moderate‐high‐very high) as well as their involvement in the organization (absent‐very low‐low‐moderate‐high‐very high). Moreover, they were enquired about their position in the organization (president or other board member—employed by the organization‐voting member—non‐voting but active member and non‐active member) and their membership duration.

#### 2.2.3 Organizational characteristics

One section of the questionnaire recorded information about the CPO characteristics. In particular, respondents had to indicate whether their CPO provides information material to its members (yes‐no) and training (yes‐no). Additionally, they were asked whether their CPO was a member of a national cancer federation (yes‐no), of a national federation of chronic diseases (yes‐no) and of a national federation for people with disabilities (yes‐no).

#### 2.2.4 Country grouping

To explore potential association between contextual factors and CPO participation, countries were aggregated on the grounds of their existing legislation. Specifically, based on information emanating from the European Health Consumer Index,^47^ countries were grouped with respect to the degree to which their health‐care law is based on patient rights (low‐medium‐high).

It merits noting that the research instrument was in the native language of each country.

### 2.3 Procedure

As already described in the Sample section, a sampling frame/list consisting of existing CPOs in Europe was composed. Contacts with these organizations were made through email or telephone. Specifically, an invitation was sent out to the president of the organization or a board member. After initial acceptance of participation, the Institutional Review Board of each organization approved the study protocol. The board members of each CPO were asked to distribute the questionnaire to their members. If a member agreed to participate, he/she signed the written informed consent and was referred to the online survey link. Data were gleaned online.

The present work was conducted with the valuable input of European CPOs delegates such as the European Patient Advocates Leadership Council in Oncology.

### 2.4 Statistical analysis

Continuous variables are presented with means and standard deviations. Categorical variables are presented with absolute and relative frequencies. Hierarchical multiple regression analyses were performed with respect to each of the dependent variables: the HDI Degree and Impact subscales. Log transformations were used for the regression analyses. Three set of variables were used in the analyses: individual, organizational and country characteristics. Coefficients of determination (adjusted *R* ^2^) were reported as a measure of variation explained by the *model and standardized regression coefficients as a measure of the effect of independent variables*. All *P* values reported are two‐tailed. Statistical significance was set at 0.05, and analyses were conducted using SPSS statistical software (version 19.0).

## 3 RESULTS

### 3.1 Sample characteristics

The majority of respondents were women (57.8%) and their mean age was 54.34 years old. Regarding their educational attainment, the majority of participants had completed undergraduate studies (41.1%), while a substantial proportion had also completed postgraduate studies (27.8%). Concerning their position in the organization, most of the participants stated that they are non‐voting but active members (36.8%), while 1 of 4 was the president of the organization or other board member (25.3%). Moreover, the majority of respondents were patients (42.4%), while a noteworthy proportion was relatives of people with cancer (33.1%). The mean membership duration was found to be 6.79 years and most of the respondents rated their involvement in the organization as moderate (32.4%) to high (32.5%). Sample characteristics are presented in detail in Table 1.

**Table 1:** Sample characteristics

	Ν (%)	Mean (SD)
Gender		
Male	534 (42.2)	
Female	732 (57.8)	
Educational level		
Νon‐formal qualification	9 (0.7)	
Primary school education (up to age 12)	7 (0.6)	
Secondary school education (up to age 15‐16)	74 (5.8)	
Secondary school education (up to age 18)	304 (24.0)	
University degree	520 (41.1)	
Postgraduate degree	352 (27.8)	
Age		54.34 (10.09)
Position in the organization		
President or other board member	320 (25.3)	
Employed by the organization	161 (12.7)	
Voting member	256 (20.2)	
Non‐voting but active member	466 (36.8)	
Non‐active member	63 (5.0)	
Personal involvement in the organization		
Νone	14 (1.1)	
Very low	28 (2.2)	
Low	68 (5.4)	
Moderate	410 (32.4)	
High	412 (32.5)	
Very high	334 (26.4)	
Membership duration (in years)		6.79 (6.28)

### 3.2 Degree and Impact of CPO participation

As illustrated in Table 2, the highest degree of CPO participation was observed with regard to hospital boards (mean = 6.21, SD = 1.3), in reforms or key decisions in health policy (mean = 5.36, SD = 1.52) and in ethics committees for clinical trials (mean = 5.27, SD = 1.67). On the contrary, participation in panels/workshops in other important health‐related organizations (mean = 4.28, SD = 1.59) and in the Ministry of Health (mean = 4.37, SD = 1.62) were found to have the lowest mean values.

**Table 2:** Item descriptives for the HDI degree subscale

	Mean (SD)
Does your patient organization participate	
In reforms or key decisions in health policy	5.36 (1.52)
In panels of experts or workshops held in the Ministry of Health	4.37 (1.62)
In panels or workshops in other important organizations pertinent to health	4.28 (1.59)
In hospital boards	6.21 (1.30)
In ethics committees for clinical trials	5.27 (1.67)
In health technology assessment procedures for the scientific evaluation of new treatments and methods	4.49 (1.75)
In health technology assessment procedures for the economic evaluation of new treatments and methods	4.45 (1.76)
In the national parliament during decision making for important health policies/legislation	5.07 (1.85)
HDI score (sum)	24.59 (9.49)

Interestingly, the reverse pattern of results was observed with regard to the impact dimension of the HDI (Table 3). In particular, the highest impact was observed with respect to participation in panels/workshops at the Ministry of Health (mean = 3.27, SD = 1.18) and other important health‐related organizations (mean = 3.24, SD = 1.25), while the lowest scores were discerned with regard to participation in hospital boards (mean = 1.97, SD = 1.17), in the national parliament (mean = 2.51, SD = 1.18) and in the ethics committees (mean = 2.46, SD = 1.24). Similarly, a low score was found for the item enquiring about the frequency whereby a substantial change in the content of a health policy decision occurs as a corollary of CPO involvement (mean = 2.59, SD = 1.27).

**Table 3:** Item descriptives for the HDI impact subscale

	Μean (SD)
How would you rate the outcome (impact) of this participation	
In reforms or key decisions in health policy	2.72 (1.30)
In panels of experts or workshops held in the Ministry of Health	3.24 (1.25)
In panels or workshops in other important organizations pertinent to health	3.27 (1.18)
In hospital boards	1.97 (1.17)
In ethics committees for clinical trials	2.46 (1.24)
In health technology assessment procedures for the scientific evaluation of new treatments and methods	2.93 (1.31)
In health technology assessment procedures for the economic evaluation of new treatments and methods	2.75 (1.30)
In the national parliament during decision making for important health policies/legislation	2.51 (1.18)
How often do you observe a substantial change in the content of a health policy decision as a result of the involvement of your patient organization?	2.59 (1.27)
HDI score (sum)	24.60 (7.82)

It is noteworthy that mean values in the Degree subscale were conspicuously higher than the corresponding in the Impact subscale.

### 3.3 Correlates of CPO participation

When multiple regression analysis was conducted with the HDI Degree subscale as the dependent variable (Table 4), the position in the organization and membership duration were found to be independently associated with the HDI Degree composite score. Concerning organization characteristics, the CPO being a member of a national cancer federation or a member of a national federation for chronic diseases was predictive for a higher score on the subscale. Additionally, both the “medium” and “high” country categories were found to have higher HDI Degree scores as compared to the “low” country category. Country aggregation, personal position in the organization and the CPO being a member of a national cancer federation or a member of a national federation for chronic diseases were found to bear the strongest associations with the HDI subscale scores, as evidenced by the standardized regression coefficients. Both organizational and country characteristics increased the *R* ^2^ of the model, thus indicating that an additional proportion of variance was explained by the aforementioned factors.

**Table 4:** Multivariate regression analysis for the HDI degree subscale

	β	SE	β*	*P*	*R* ^2^
Personal characteristics					
Rate your familiarity with the disease	−0.003	0.01	−0.02	.714	.06
Rate your knowledge about treatment options/country's health‐care system/country's reimbursement processes	−0.001	0.01	−0.02	.591	
What is your position in the organization?	0.05	0.01	**0.34**	**<.001**	
How long in years have you been a member?	0.002	0.001	0.09	**.002**	
Rate your personal involvement in the organization	−0.002	0.01	−0.01	.745	
Organization characteristics					
I receive information materials	−0.004	0.01	−0.01	.733	.16
I receive training	0.01	0.01	0.01	.724	
My PO is a member of a national cancer federation	0.08	0.01	**0.21**	**<.001**	
My PO is a member of a national federation for chronic diseases	0.07	0.01	**0.20**	**<.001**	
My PO is a member of a national federation for people with disabilities	−0.01	0.02	−0.02	.580	
Country characteristics					
Health‐care law is based on patient rights					
Low, reference					
Medium	0.15	0.02	**0.22**	**<.001**	.29
High	0.24	0.02	**0.49**	**<.001**	

Bold values is smaller than *P* < .001.

β = regression coefficient; SE = standard error; β* = standardized regression coefficient; *R* ^2^ = coefficients of determination.

Multiple regression analysis for the HDI Impact subscale (Table 5) revealed that among personal characteristics position in the organization and personal involvement in the organization had a significant association. The addition of organization and country characteristics on the block of variables concerning personal characteristics increased the *R* ^2^ of the model, thus indicating that an additional proportion of variance was explained by the model. Specifically, among organization characteristics, receiving information materials or training as well as the CPO being a member of a national cancer federation or a member of a national federation for chronic diseases was predictive for a higher score on the HDI Impact subscale. Also, a greater HDI Impact score was observed in the “high” country category as compared to the low category. Personal involvement in the organization, the position in the organization, receiving information materials and the CPO being a member of a national cancer federation or of a national federation for chronic diseases had the strongest association with the HDI Impact subscale score, as evidenced by the standardized regression coefficients.

**Table 5:** Multivariate regression analysis for the HDI impact subscale

	β	SE	β*	*P*	*R* ^2^
Personal characteristics					
Rate your familiarity with the disease	0.01	0.01	0.05	.248	0.08
Rate your knowledge about treatment options/country's health‐care system/country's reimbursement processes	0.001	0.01	0.01	.880	
What is your position in the organization?	0.02	0.004	**0.15**	**<.001**	
How long in years have you been a member?	−0.001	0.001	−0.02	.441	
Rate your personal involvement in the organization	0.04	0.01	**0.25**	**<.001**	
Organization characteristics					
I receive information materials	0.05	0.01	**0.19**	**<.001**	0.18
I receive training	0.02	0.01	0.07	**.018**	
My PO is a member of a national cancer federation	0.03	0.01	**0.12**	**<.001**	
My PO is a member of a national federation for chronic diseases	0.04	0.01	**0.14**	**<.001**	
My PO is a member of a national federation for people with disabilities	0.001	0.01	0.003	.918	
Country characteristics					
Health‐care law is based on patient rights					
Low, reference					
Medium	−0.03	0.02	−0.05	.165	0.21
High	0.03	0.01	0.08	**.033**	

Bold values is smaller than *P* < .001.

β = regression coefficient; SE = standard error; β* = standardized regression coefficient; *R* ^2^ = coefficients of determination.

## 4 DISCUSSION

The present study is the first endeavour to systematically investigate the degree and impact of CPO participation in health policy across Europe. To this end, a previously developed instrument assessing the degree and impact of PO participation in various realms of decision making at the meso‐ and macro‐level (regional, national and international) was employed.

Study findings indicate that a higher degree of patient participation is not necessarily translated into a higher impact. Arguably, the two are interlinked; however, a different pattern of results—in fact the reverse—emerged between the items of the Degree and the Impact subscale. Concomitantly, the multivariate linear models revealed both similarities and differences in the correlates of the two subscales, suggesting that the two dimensions are interwoven but should not be considered identical. In other words, a high degree of patient organization participation does not guarantee the effectiveness of this participation. A similar observation has been mooted by the handful of qualitative studies in the field.^12^, ^23^, ^24^, ^25^, ^26^, ^27^, ^28^, ^29^, ^30^, ^33^, ^34^ Evidence from UK, USA, Germany, Italy and the Netherlands substantiates the growing number of patient organizations in these countries as well as their heightened involvement in policy processes; however, the impact of their participation is disproportionately low.^12^, ^23^, ^24^, ^25^, ^26^, ^27^, ^28^, ^29^, ^30^, ^33^, ^34^ This echoes the concern raised by some authors that governments may capitalize on patient participation for adding legitimacy to their own interests,^30^ thus resulting to what appears to be ostensible participation. A similar concern about the distinction between representation and representativeness has long been raised by Angela Coulter,^48^ who has highlighted the dangers of tokenism entailed in patient representation committees, when they are not sufficiently independent from trust management in the UK.

This distinction between degree and impact of participation, as tapped by the HDI scale, can also be viewed in the light of the difference between the so‐called input democracy and output democracy.^49^ Sarah Hobolt draws upon the seminal quote of A. Lincoln on democracy: that is “government by the people, of the people and for the people”.^50^ In her stream of argument, input democracy refers to the procedures that allow citizen participation and input into the democratic process—the “by the people” clause, whereas output democracy stresses government effectiveness and performance—the “for the people” clause.^50^ In this regard, findings from the present study raise concerns about the possibility of an output (health) democracy deficiency in EU‐28.

The correlates of CPO participation (degree and impact) shed light on potential ways for tackling hindrances and thus facilitating patient participation in health policy. As regards the degree of CPO participation, the aggregation of countries on the grounds of a health‐care law promoting patient rights was found to be the most important correlate, as indicated by the marked rise in *R* ^2^ value, when this block of variables was included into the model, as well as by the highest standardized coefficient of the variable “high” (ie the health‐care law is based on patient rights to a high degree). Even the “medium” category appears to confer a benefit, as it increased the degree of patient participation substantially as compared to the low category. In this reasoning, legislation is an important element for securing patient participation. Of secondary importance are the organizational characteristics. Among them, coalition among CPOs or among patient organizations for chronic diseases was found to contribute markedly to enhancing the degree of CPO participation. Interestingly, with respect to the Impact subscale, the organizational characteristics emerged as the most crucial variables, as indicated by changes in *R* ^2^ values and their standardized coefficients. Umbrella organizations and the provision of information and training to members can improve the impact of CPO. Of secondary importance is the existence of a health‐care law based on patient rights. In this rationale, pertinent legislation is a prerequisite, a necessary condition, for ensuring patient participation; however, it is not sufficient, as it cannot guarantee a high impact. Therefore, countries with low levels of participation should concentrate on reforming existing health‐care legislation, rendering it more patient‐centric, while countries with low impact of participation should focus on their CPOs.

The importance of a health‐care law promoting patient rights and empowerment has long been recognized across Europe. According to the “Ljubljana Charter on Reforming Health Care in Europe”,^51^ patients have the right to participate in shared decision making on an equal basis and contribute to all relevant procedures that affect population' health, while the Council of Europe underscores that patient empowerment is an area of high priority, so as to ensure that the patient is at the centre of the health care and has access to each stage of health policy decision making.^52^ Nonetheless, the conclusion of the present study that pertinent legislation does not guarantee high impact of CPO participation concurs with the study by van de Bovenkamp and colleagues in the Netherlands.^12^ Specifically, the authors stressed that while there were too many opportunities for patient organizations to participate, many of them could not withstand the pressure. In other words, while the system in the Netherlands does not deny access to patient organizations, they often lack the resources to exert an influence. This observation fits neatly with the preponderance of coalitions and the provision of information and training in the multivariate models of the present study, especially with regard to the Impact subscale. The study by Wood^27^ suggests that in both UK and USA, there are hundreds of separate voices who aspire to influence the policy agenda; however, they operate autonomously and are reluctant to collaborate. Similarly, the workshop of Vienna^37^ has documented tensions among organizations, which are often consumed by heightened competition for media attention, membership and funding. Interestingly, this issue is intensified in the cancer realm, where the complexity and diversity of groups has hampered joint campaigning and lobbying.^30^ It merits noting All‐Party Group on Cancer Ian Gibson had called for cancer organizations to lobby altogether for better services.^30^

Concerning the provision of information and training to CPO members, the present study, mainly due to its design (quantitative study) and objectives, could not get into depth about which type of training underpins the strong independent effect of the variable on the impact of participation subscale. Based on existing literature, converging evidence indicates that providing information and thus increasing patients' health literacy levels is pivotal for enhancing patient participation, at least at the individual level.^14^ To this end, a number of patient organizations provide information materials targeting their members' health literacy levels. Similarly, with regard to cancer, the National Coalition for Cancer Survivorship has developed the Cancer Survival Toolbox for teaching self‐advocacy skills to cancer survivors, defined as the ability to obtain and understand information, to find appropriate resources and treatment modes, to get different medical opinions and to make informed choices about treatment, including the right to no treatment.^53^ On the meso‐ and macro‐level, institutions in collaboration with patient organizations have developed and delivered various training programmes on methods of evidence‐based medicine, advocacy and health‐care policy.^54^, ^55^, ^56^, ^57^, ^58^, ^59^, ^60^ On the realm of cancer, for several years EUROPA DONNA has provided advocacy training for its members, while the European Cancer Patient Coalition organizes every year a Masterclass in Advocacy.^61^ While there is currently an objection to the importance of training and thus professionalizing patient representatives to solve problems in participation practices, as this could reduce their legitimacy as representing “true” patients,^6^ evidence from our study provides evidence for the contrary. Arguably, without some degree of training delivered to patient representatives, health policymakers may use the sophisticated knowledge often required in certain areas of health policy as a pretext for excluding patient organizations from important decisions. There is some promising evidence from the Patient and Community Engagement Research Program, where the uptake of new patient roles in health‐care planning was found to start influencing attitudes and practices.^54^ Nonetheless, only a limited number of studies have evaluated the impact of these training programmes, highlighting the imperative need for future work along this research path.^60^ A further qualitative study from our research group will endeavour to collect more information about the type of training that yields a substantial impact of CPO participation on health policy. It merits noting that a number of patient organizations deliver training to health professionals as well, with this joint focus yielding a better outcome.^14^

Therefore, the establishment of a health‐care law based on patient rights as well as the formation of coalitions and the endowment of CPOs appears the most promising routes for advancing CPO participation in health policy in Europe.

### 4.1 Limitations

This is the first large‐scale quantitative study to systematically investigate this issue; however, it has certain limitations that warrant consideration. In the absence of a sampling frame for CPOs in Europe, the representativeness of the present sample and thus the generalizability of the study findings cannot be ensured. In this rationale, the external validity of the study may be a shortfall. At the same time, in spite of systematic efforts to recruit respondents from different sources, it is assumed that the most active and motivated CPO members participated in the study. As a result of this, responders may systematically differ from non‐responders and sampling bias may have arisen in the study. A future study should concentrate on constructing an adequate sampling frame to facilitate rigour in the field. Additionally, countries were grouped in terms of legislation. An alternative way would have been to take into account characteristics of the health‐care system. A future analysis from our research group will try to address this alternative strategy of analysis.

It is noteworthy that findings of the present study are constrained to cancer patient organizations and cannot be extrapolated to other health conditions. While the literature suggests substantial variation among disease groups with respect to patient participation,^16^, ^43^ no study to date has aimed at directly addressing this objective. Some evidence on patient empowerment indicates that empowerment exerts no impact on therapy compliance of patients with most severe diseases, such as cancer, whereas it enhances therapy compliance among those with less severe conditions, such as diabetes.^43^ At the collective level, the De Montfort study in UK suggested that cancer and mental health groups were well represented in influencing policy, as compared to other disease groups; however, it did not specifically assess the impact on health policy as a function of disease group.^16^ Therefore, research on patient participation should take into consideration differences among disease groups. By utilizing the HDI, a further study may directly set out to examine this issue.

It merits noting that among the strengths of the present study is that data were based on responses made primarily by patients and their relatives (75.5%). However, the study could not have gone into depth with regard to the internal dynamics of CPOs and more specifically the degree of internal democracy in these organizations. It has long been posited that a dearth of internal participatory practices may result in elite decision making and a misrepresentation of interests within the organization.^62^ Evidence from the De Montfort study indicates that the same people—disparagingly called as “usual suspects”—tend to appear as representatives of health consumer groups, while existing formal and informal internal participatory practices (such as elections to office and annual general meetings) within patient organizations have not been systematically explored.^30^ Concomitantly, the government appears oblivious about how group representativeness has been achieved and it is likely in some cases that leaders may misrepresent their membership or that a group may be controlled by professional or commercial interests. In this regard, a further study—preferably a qualitative one to allow for more in‐depth responses—should aim to systematically explore members' views about their leaders and internal participatory practices within organizations.

In conclusion, quantitative research on CPO participation in EU‐28 indicates that a high degree of CPO participation does not necessarily lead to high impact of CPO participation, raising concerns about an output health democracy deficiency in Europe. Initiatives to enhance the former should be geared towards promoting the establishment and enforcement of a health‐care law based on patient rights. In addition, the formation of umbrella organizations and the provision of information and training on CPO members may foster the political effectiveness of the organizations.

## CONFLICT OF INTEREST

Research was funded by Novartis Pharma, Basel, under the AGORA initiative—a European Think Tank which aims to optimize patient access to innovative treatments. Nonetheless, the company was not involved in any way in the design, implementation and interpretation of research findings.
